# Association between sleep-disordered breathing and periodontitis: a meta-analysis

**DOI:** 10.4317/medoral.25627

**Published:** 2022-12-24

**Authors:** Xinyu Liu, Zhenkun Zhu, Peng Zhang

**Affiliations:** 1 Department of Oral Medicine, Qilu Hospital of Shandong University, Jinan, China; 2 School and Hospital of Stomatology, Cheeloo College of Medicine, Shandong University and Shandong Key Laboratory of Oral Tissue Regeneration and Shandong Engineering Laboratory for Dental Materials and Oral Tissue Regeneration, Jinan, China; 3 Department of orthopedics, The Affiliated Hospital of Shandong University of Traditional Chinese Medicine, Jinan, China

## Abstract

**Background:**

Systemic inflammation is a feature of sleep-disordered breathing (SDB) as well as periodontitis. The association between SDB and periodontitis, however, has been inconsistent in previous studies. In order to fully evaluate the above association, we conducted a meta-analysis.

**Material and Methods:**

Observational studies related to the aim of the meta-analysis were identified by search of PubMed, Embase, Web of Science, Wanfang, and CNKI databases. Only studies with SDB diagnosed with the objective polysomnography examination were included. The results were analyzed using a random-effects model that incorporated potential heterogeneity between studies.

**Results:**

Ten cross-sectional or case-control studies with 43,296 participants contributed to the meta-analysis. Pooled results showed that SDB was significantly associated with periodontitis (odds ratio [OR]: 1.83, 95% confidence interval [CI]: 1.52 to 2.20, I2 = 40%, *p* < 0.001). Sensitivity analysis showed consistent association for severe periodontitis (OR: 1.39, 95% CI: 1.20 to 1.61, I2 = 0%, *p* < 0.001). Subgroup analyses showed consistent results in patients with mild (OR: 1.66, *p* < 0.001), moderate (OR: 2.23, *p* = 0.009), and severe SDB (OR: 2.66, *p* < 0.001). Moreover, the association between SDB and periodontitis was consistent in Asian and non-Asian studies, in cross-sectional and case-control studies, in studies with univariate and multivariate regression models, and in studies with different quality scores (*p* for subgroup effects all < 0.05).

**Conclusions:**

Polysomnography confirmed diagnosis of SDB is associated with periodontitis in adult population.

** Key words:**Sleep-disordered breathing, periodontitis, cross-sectional studies, hypoxia, meta-analysis.

## Introduction

The inflammation of periodontal tissues causes periodontitis, a chronic oral disease characterized by destruction of multiple tissues that support teeth, such as cement, periodontal ligament and alveolar bone (1). The main initial pathological change of periodontitis is the chronic bacterial infection of the tooth supporting structures, and the continuous inflammation in the periodontal tissues could lead to various consequences, from gingival bleeding to tooth loss (1). The prevalence of periodontitis in the global population is suggested to be more than 50% (2), and periodontitis has become one of the most severe and prevalent oral disorders which significantly impair the quality of life of the patients (3). Accordingly, for early prevention and treatment of periodontitis, it is critical to identify the clinical factors that contribute to its pathogenesis.

Previous studies have revealed many factors that are associated with the risk of periodontitis, such as aging (4), obesity (5), smoking (6), alcohol consumption (7), and diabetes (8). Besides, accumulating evidence suggests that various inflammatory diseases are associated with higher odds of periodontitis, such as rheumatoid arthritis (9), systemic lupus erythematosus (10), Crohn's disease (11) and ulcerative colitis etc. (12), suggesting that systemic inflammation may be an important determinant of periodontitis (13). The sleep-disordered breathing (SDB) syndrome is characterized by recurrent episodes of intermittent pauses or reductions of breathing during sleep, resulting in hypoxia (14). Clinically, SDB is diagnosed using polysomnography (PSG) with the measurement of apnea-hypopnea index (AHI) (15). The pathological features of SDB include intermittent hypoxia, oxidative stress, and chronically activated inflammatory response (16), which have also been involved in the pathogenesis of periodontitis. Moreover, SDB and periodontitis shared a few common risk factors, such as aging, obesity, and smoking (16), which collectively suggests that SDB may be associated with periodontitis. It is unclear, however, whether SDB is associated with periodontitis in adults in previous studies (17-26). Some studies suggested that SDB may be a risk factor of periodontitis (17-20,24,25), while others did not (21-23,26). The aim of this study was to assess the association between SDB and periodontitis in an adult population by means of a meta-analysis.

## Material and Methods

This systematic review and meta-analysis were conducted according to the Preferred Reporting Items for Systematic Reviews and Meta-Analyses (PRISMA) statement (27) and the Cochrane’s Handbook (28) guidelines.

- Database search

Studies were retrieved by search of the electronic databases including PubMed, Web of Science, Embase, Wanfang, and CNKI databases from inception to March 26, 2022, with a combined search terms of 1) "obstructive sleep apnea syndrome" OR "sleep apnea syndrome" OR "obstructive sleep apnea" OR "obstructive sleep hypopnea syndrome" OR "sleep disordered breathing" OR "sleep breathing disorders" OR "OSAHS" OR "OSAS"; and 2) "periodontal" OR "periodontitis" OR "oral health". There was no restriction on the publication language, only human studies were searched. A manual screening of references from relevant original and review articles was also conducted. During the meta-analysis, only full-length articles published in peer-reviewed journals were included.

- Study inclusion and exclusion criteria

A PICOS-recommended set of inclusion criteria was developed based on the meta-analysis's purpose.

P (participants): Adult population (18 years old or above).

I (exposure): Patients with SDB as diagnosed with the objective PSG examination.

C (control): Patients without SDB.

O (outcomes): Odds ratio (OR) of periodontitis between patients with and without SDB. Periodontitis was diagnosed according to the findings of the periodontal examination.

S (study design): Observational studies, which include case-control studies, cross-sectional studies, and cohort studies.

Reviews, meta-analyses, editorials, studies including children, studies with SDB diagnosed using the self-reported questionnaires, or studies that did not report the outcome of periodontitis were excluded. When there was overlap in the population of two studies, we included the study with the largest sample size.

- Data collection and quality assessing

During the research process, two authors independently analyzed literature, collected data, and assessed the quality of the study. Discrepancies were discussed with the corresponding author if they occurred. We extracted data regarding basic study information, participant characteristics, age, sex, methods for the diagnosis of SDB, number of patients with SDB, methods for the diagnosis of periodontitis, number of patients with periodontitis, and variables adjusted when the association between SDB and periodontitis was presented. As a measure of study quality, Newcastle-Ottawa scales (NOS) were used (29), on the basis of participant selection criteria, group comparison, and outcome validity. A study's quality is assessed on a scale of 1-9 stars, with a higher number of stars indicating a higher standard of study.

- Statistics

The association between SDB and periodontitis in adult population was presented as OR and the 95% confidence interval (CI). A meta-analysis was performed on the OR data derived with the most appropriately adjusted model in studies analyzing the above association. By using the 95% confidence intervals or *p* values, ORs and standard errors (SEs) may be calculated. We then transformed the distribution using logarithms in order to maintain stabilized variances and normalized distributions. Heterogeneity between studies was determined using Cochrane's Q test and I2 statistics (28). The between-study heterogeneity was classed as mild (I2 < 25%), moderate (I2 25%~75%), and high (I2 >75%) according to the Cochrane’s Handbook (28). The results were combined using a random-effects model incorporating heterogeneity's influence (28). Meta-analysis results were evaluated by excluding one dataset at a time to determine how individual studies influenced the results (28). Sensitivity analysis was performed to evaluate the association between SDB and severe periodontitis, which was defined according to the American Academy of Periodontology and the Centers for Disease Control and Prevention (AAP/CDC) definitions of periodontal disease (30). Subgroup analyses according the SDB severity, study country, study design, regression model, and study quality scores were also performed. The severity of SDB was defined with usual clinical thresholds with AHI, which could be classified as mild (AHI ≥ 5 to < 15 events per hour), moderate (AHI ≥ 15 to < 30 events per h), and severe (AHI ≥ 30 events per hour) (15). An estimation of publication bias was performed using funnel plots constructed by visual judgement of symmetry, with an Egger's regression asymmetry test in addition (28). The RevMan (Version 5.1; Cochrane Collaboration, Oxford, UK) and Stata software (version 12.0; Stata Corporation, College Station, TX) were used for the statistical analyses.

## Results

- Literature search

**Figure 1:**
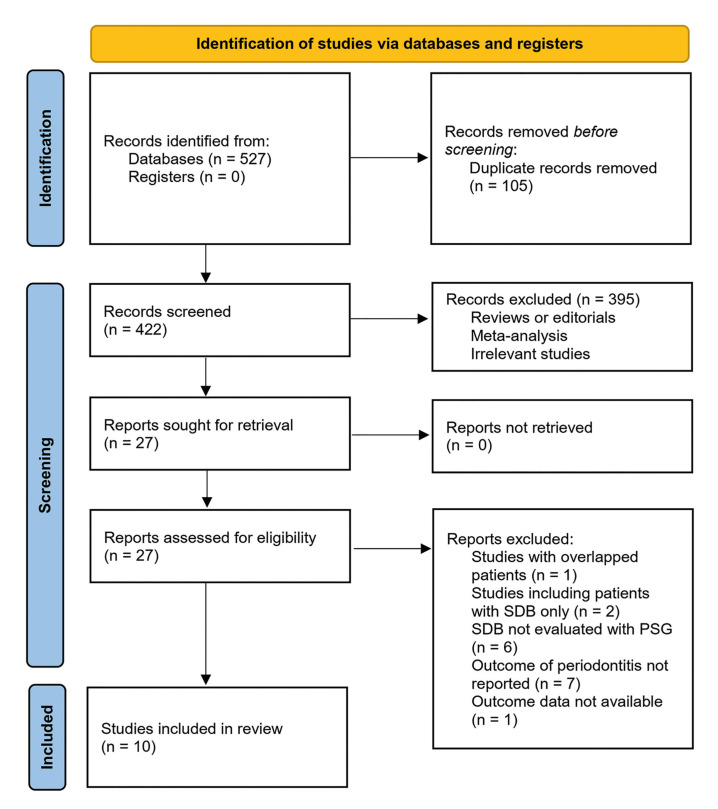
Diagram of database search and study inclusion.

Fig. 1 shows the literature search and inclusion process. Overall, 527 records were obtained from the initial database search, with 105 being removed due to duplication. Three previous meta-analyses (31-33) were obtained during the literature search, and were discussed subsequently. After screening titles and abstracts of 422 studies, 395 were excluded largely due to non-relevance to the meta-analysis's objective according to the predefined inclusion criteria. Ultimately, 27 studies were reviewed in full-text, and 17 were excluded for the reasons listed in Fig. 1, leading to 10 studies available for the meta-analysis.

- Study characteristics

Table 1 shows characteristics of the studies included. Overall, two case-control studies (17,26) and eight cross-sectional studies (18-25) contributed to the meta-analysis. These studies were performed in China (17,24,25), Korea (18), the United States (19,20), Turkey (21,22), Columbia (23), and Spain (26), and published between 2013 and 2021. A total of 43,296 adults were included, and the mean ages of the patients varied between 29 and 56 years. For all of the included studies, PSG was used to for the diagnosis of SDB, and 11,773 (27.2%) of the participants were diagnosed as SDB. Periodontitis was diagnosed according to the findings of the periodontal examination for all of the included studies, and the diagnosis of periodontitis was in accordance with the AAP/CDC definitions in eight of the studies (18-23,25,26). Specifically, according to the AAP/CDC definitions (34), mild periodontitis was defined as ≥ 2 interdental sites with clinical attachment level (CAL) ≥ 3 mm and ≥ 2 interdental sites with probing depth (PD) ≥ 4 mm (not in the same tooth), or one site with PD ≥ 5 mm. Moderate periodontitis was defined as ≥ 2 interdental sites with CAL ≥ 4 mm (not in the same tooth), or ≥ 2 interdental sites with PD ≥ 5 mm. Lastly, severe periodontitis was defined as ≥ 2 interdental sites with CAL ≥ 6 mm (not in the same tooth) and ≥ 1 interdental site with PD ≥ 5 mm. For the other two studies, patients with the positive findings of periodontal examination who received either deep cleaning or a surgical procedure for the treatment of periodontitis were diagnosed as periodontitis in one study (17), and patients with ≥ 2 interdental sites with CAL ≥ 3 mm or ≥ 2 interdental sites with PD ≥ 4 mm were diagnosed as periodontitis in the other study (24). Accordingly, 9,132 (21.1%) participants were diagnosed as periodontitis. Possible confounding factors such as age, sex, body mass index, smoking, alcohol use, and comorbidities were adjusted to a varying degree in six studies (17,18,20,22,25,26) when the association between SDB and periodontitis was analyzed, while for the other four studies (19,21,23,24), univariate analyses were used. Studies included in this review received a total of seven to nine stars according to the NOS, suggesting a generally high level of study quality (Table 2).

- Meta-analysis results

**Figure 2:**
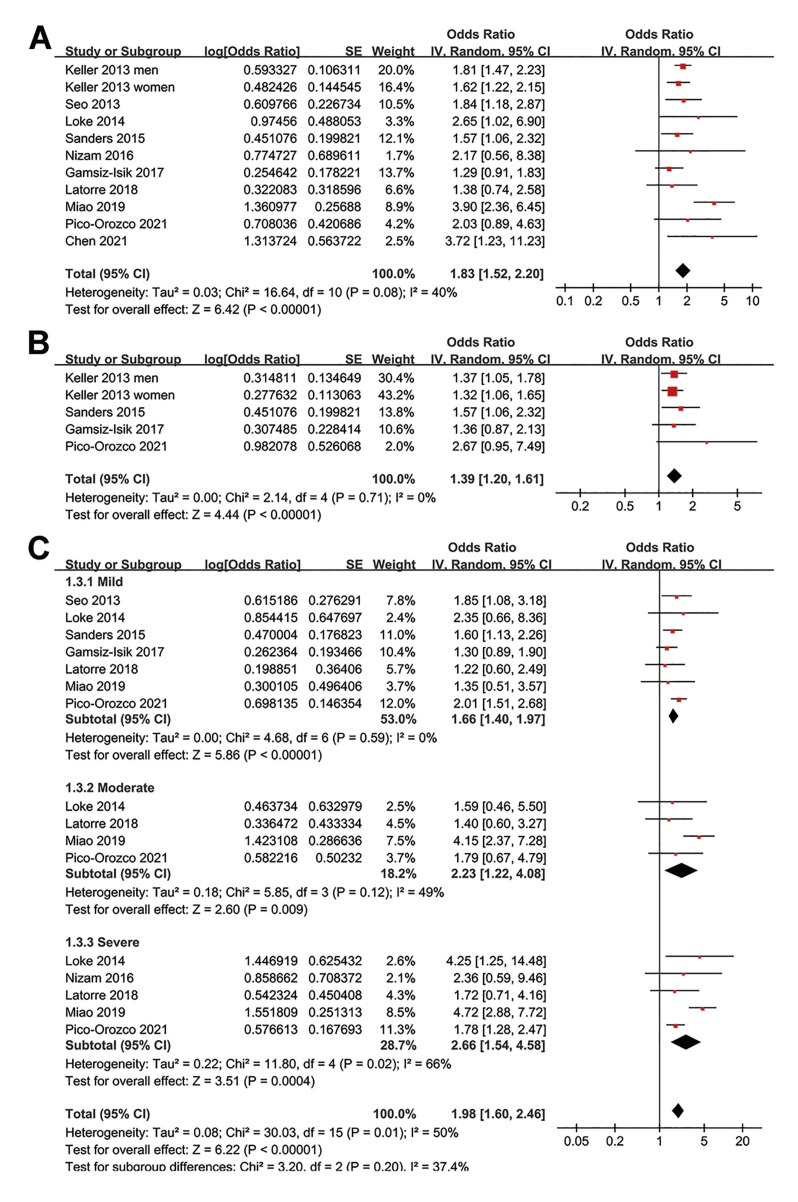
Forest plots for the meta-analysis regarding the association between SDB and periodontitis in adult population; A, overall meta-analysis; B, sensitivity analysis for the association of between SDB and severe periodontitis; C, subgroup analysis according to the severity of SDB.

Since one study reported the association between SDB and periodontitis in men and women separately (17), these datasets were independently included in the meta-analysis. According, 11 datasets from 10 studies were available for the meta-analysis. Pooled results showed that SDB was significantly associated with periodontitis in adult population (OR: 1.83, 95% CI: 1.52 to 2.20, *p* < 0.001; Fig. 2) with moderate heterogeneity (*p* for Cochrane’s Q test = 0.08, I2 = 40%). Sensitivity analysis showed consistent association for severe periodontitis (OR: 1.39, 95% CI: 1.20 to 1.61, *p* < 0.001; Fig. 2) with no significant heterogeneity (I2 = 0%). Influencing analysis by excluding one dataset at a time did not significantly affect the results (OR: 1.68 to 1.92, *p* all < 0.05).

**Table 1:**
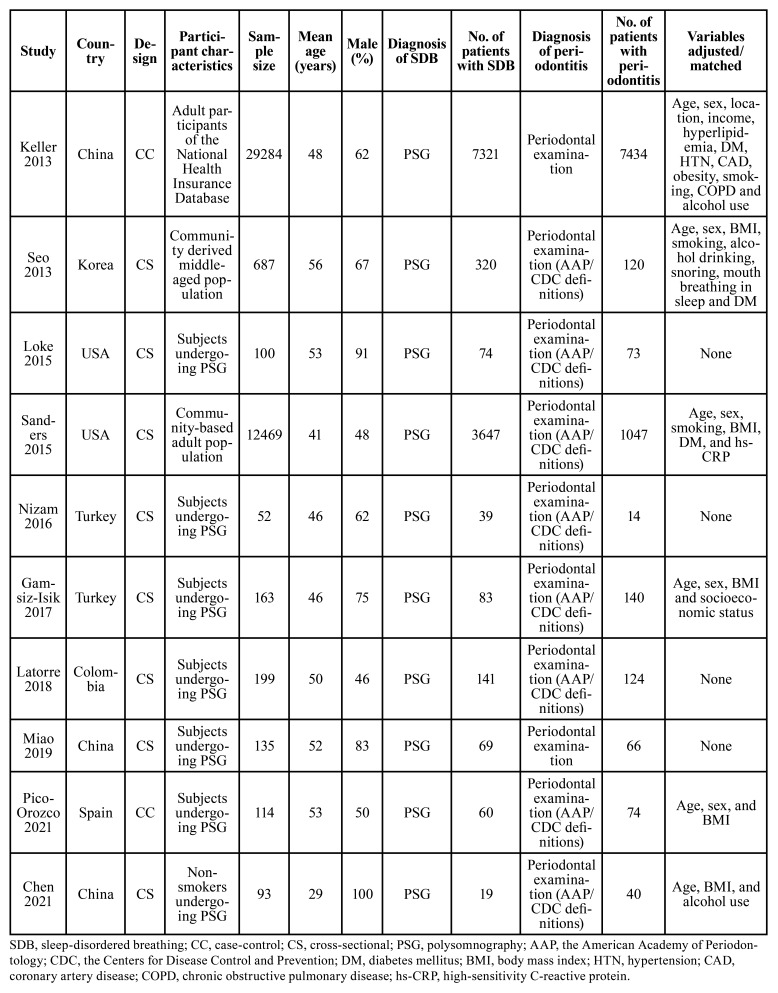
Main characteristic of the included studies.

**Table 2:**
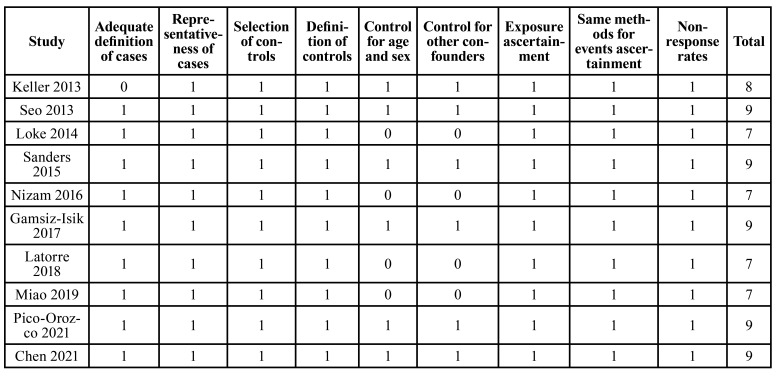
Study quality evaluation via the Newcastle-Ottawa Scale.

Further subgroup analyses according to the severity of SDB showed that the association between SDB and periodontitis was consistent for mild (OR: 1.66, 95% CI: 1.40 to 1.97, *p* < 0.001; I2 = 0%), moderate (OR: 2.23, 95% CI: 1.22 to 4.08, *p* = 0.009; I2 = 49%), and severe SDB (OR: 2.66, 95% CI: 1.54 to 4.58, *p* < 0.001; I2 = 66%; Fig. 2).

**Figure 3:**
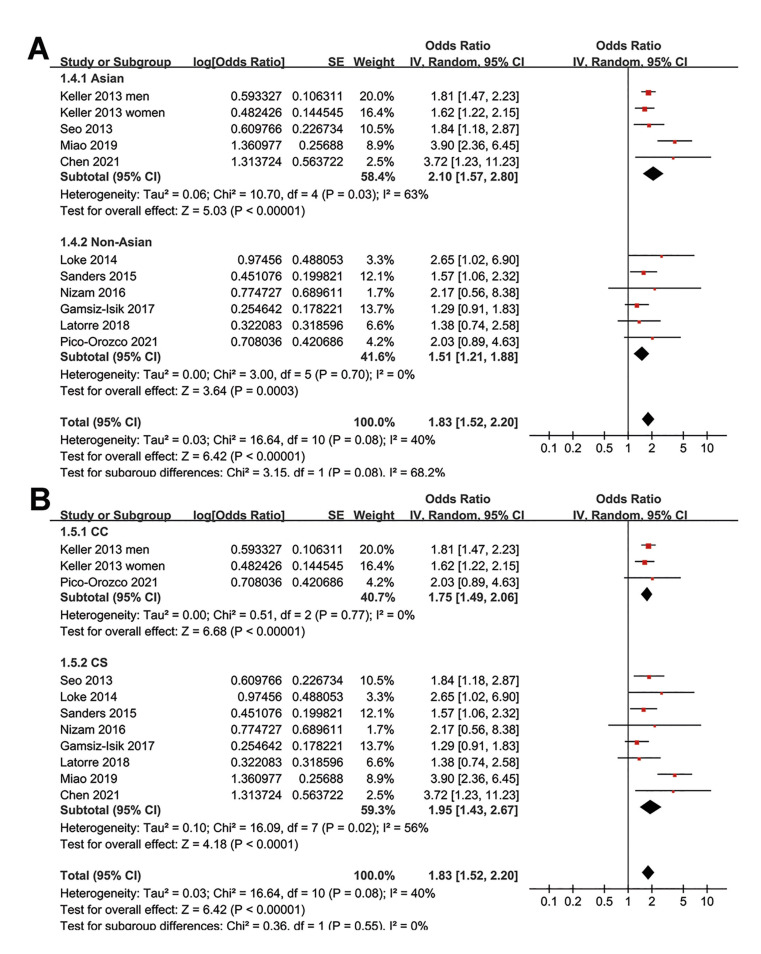
Forest plots for the subgroup analyses of the association between SDB and periodontitis in adult population; A, subgroup analysis according to the study country; and B, subgroup analysis according to the study design.

**Figure 4:**
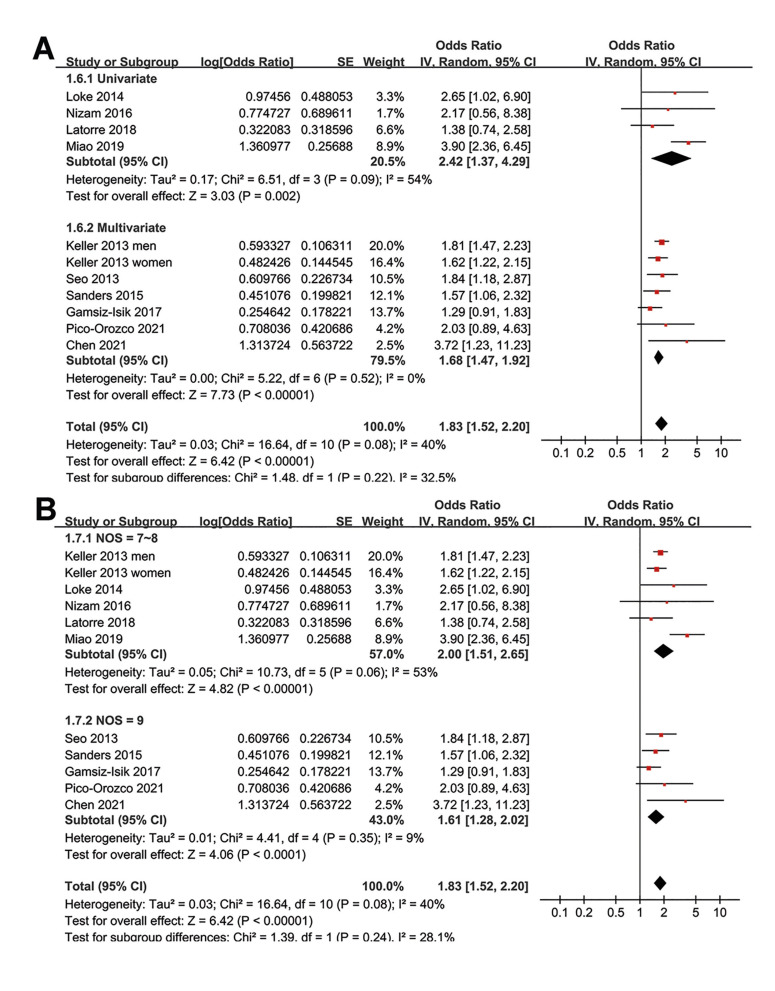
Forest plots for the subgroup analyses of the association between SDB and periodontitis in adult population; A, subgroup analysis according to the analytic models; and B, subgroup analysis according to the study quality scores.

Moreover, the association between SDB and periodontitis was consistent in Asian and non-Asian studies (Fig. 3), in cross-sectional and case-control studies (Fig. 3), in studies with univariate and multivariate regression analyses (Fig. 4), and in studies with different quality scores (Fig. 4, *p* for subgroup effects all < 0.05).

- Publication bias

The funnel plots of SDB and periodontitis in adult populations are shown in supplement 1. According to visual inspection, the plots were symmetrical, reflecting low publication bias risk. The Egger's regression testing confirmed this (*p* = 0.34).

## Discussion

In this systematic review and meta-analysis, we integrated the results of ten observational studies, and the results showed that PSG-diagnosed SDB is associated with higher odds of periodontitis in adult population. In a further sensitivity analysis, the association between severe periodontitis and SDB was found to be consistent. Moreover, further subgroup analyses showed that the association between SDB and periodontitis were consistent in participants with mild, moderate and severe SDB, in Asian and non-Asian studies, in studies of case-control and cross-sectional design, in studies with univariate and multivariate regression analyses, and in studies with different quality scores. Taken together, these results suggest that PSG confirmed diagnosis of SDB is associated with higher odds of periodontitis in adult population. Prospective studies should be considered to determine if SDB is an independent risk factor for periodontitis in adult population.

An early meta-analysis including four observational studies published before 2014 suggested that SDB may be associated with periodontitis (31). However, in one of the included studies, diagnosis of SDB was self-reported by the participants. Compared to the gold diagnostic tool of PSG with AHI results, diagnosis of SDB on the basis of patient-reported symptoms or questionnaires such as the Berlin questionnaire and the STOP-Bang questionnaire have been found to be less reliable and efficient (35,36). Moreover, although significant heterogeneity was observed (I2 = 92%), the authors did not perform subgroup analyses to explore the source of heterogeneity. During the preparation of the manuscript, two meta-analyses of the similar topic are published very recently (32,33). These two meta-analyses, including ten observational studies published before 2021 respectively, both showed that SDB are associated with higher odds of periodontitis. However, similar to the early meta-analyses, in three of the included studies, SDB was based on self-reported symptoms or questionnaires, which may confound the results. Moreover, subgroup analyses were also not performed, and studies using univariate and multivariate analyses were pooled together, which makes the interpretation of the findings difficult. As mentioned before, SDB and periodontitis have some common risk factors, such as aging, obesity, and smoking etc., which may confound the association between SDB and periodontitis (37,38). Compared to the pervious meta-analyses, the strengths of our meta-analysis are clear. Firstly, more strict inclusion criteria were applied and only studies with SDB diagnosed with PSG were included, which therefore could avoid the influence of inaccurate diagnoses of SDB in studies with SDB diagnosed on self-reported symptoms or questionnaires. Moreover, extensive literature search in five databases were performed, which retrieved 10 up-to-date studies, with two of them published in 2021. Finally, based on multiple subgroup and sensitivity analyses, the consistent results indicated the robustness of the finding, which was not driven by either of the included studies and independent of multiple study characteristics.

Specifically, we performed subgroup analyses according to the severity of SDB, which showed the association was consistent for mild, moderate, and severe SDB. Interestingly, the ORs for the association increased gradually in patients with mild, moderate, and severe SDB (1.66, 2.23, and 2.66). Although the between-subgroup difference was not statistically significant (*p* = 0.20), these findings may suggest a possible dose-response relationship between SDB and the odds of periodontitis, which deserves to be determined in large-scale studies in the future. Besides, subgroup analyses showed that the association between SDB and periodontitis were similar in studies with univariate and multivariate analyses. This is also important because the significant finding in subgroup of multivariate studies may suggest the association between SDB and periodontitis was independent of previously proposed confounding factors, such as age, obesity, smoking, and comorbidities such as diabetes. As of now, it is generally unclear what mechanisms underlie the association between SDB and periodontitis. However, several small observational studies have showed that periodontal inflammation may be an important factor involved. For example, one of the included studies showed that compared to those without SDB, patients with mild, moderate, and severe SDB were all associated with higher salivary interleukin-6 (21), an important inflammatory cytokine involved in the regulation of host response to bacterial infection during the pathogenesis of periodontitis (39). Besides, a recent study has suggested that changes of salivary microbiome in patients with SDB may be an important mediator for the pathogenesis of periodontitis in these patients. Specifically, the authors showed the species richness and trans-habitat diversity was altered, along with an increase in *Prevotella*, a specific periodontal pathogen, in patients with SDB as compared to those with SDB (40). A deeper understanding of the underlying mechanisms behind the association between SDB and periodontitis is warranted.

Limitations also exist in our study. Firstly, all of the included studies were case-control or cross-sectional studies, and the independent risk of periodontitis from SDB needs to be confirmed in high-quality prospective studies. In addition, although subgroup with multivariate studies showed a consistent association between SDB and periodontitis, some residual factors may also exist which may confound the association, such as the potential difference of oral hygiene between patients with and without SDB. Besides, a dose-response relationship between SDB and periodontitis could not be estimated based on our findings. Studies evaluating the correlation of AHI with the risk and severity of periodontitis should be considered. Lastly, since the findings are based on observational studies, we are not able to draw a causal link between SDB and periodontitis. If prospective studies could confirm SDB as a risk factor for periodontitis, there needs to be further research to determine whether chronic positive airway pressure, an intervention for SDB, affects periodontal health.

## Conclusions

To sum up, the meta-analysis found that PSG confirmed diagnosis of SDB is associated with periodontitis among adults. It is necessary to conduct future studies to determine whether SDB is an independent risk factor for periodontitis and identify the potential underlying mechanisms.
